# Dynamic Stability of Nanobeams Based on the Reddy’s Beam Theory

**DOI:** 10.3390/ma16041626

**Published:** 2023-02-15

**Authors:** Youqin Huang, Richeng Huang, Jiachang Zhang

**Affiliations:** Research Centre for Wind Engineering and Engineering Vibration, Guangzhou University, Guangzhou 510006, China

**Keywords:** dynamic instability, nanobeam, Reddy beam theory, nonlocal theory, elastic medium, parametric analysis

## Abstract

The dynamic stability of nanobeams has been investigated by the Euler-Bernoulli and Timoshenko beam theories in the literature, but the higher-order Reddy beam theory has not been applied in the dynamic stability evaluation of nanobeams. In this work, the governing equations of the motion and dynamic stability of a nanobeam embedded in elastic medium are derived based on the nonlocal theory and the Reddy’s beam theory. The parametric studies indicate that the principal instability region (PIR) moves to a lower frequency zone when length, sectional height, nonlocal parameter, Young’s modulus and mass density of the Reddy nanobeam increase. The PIR shifts to a higher frequency zone only under increasing shear modulus. Increase in length makes the width of the PIR shrink obviously, while increase in height and Young’s modulus makes the width of the PIR enlarge. The sectional width and foundation modulus have few effects on PIR.

## 1. Introduction

For a beam under an axially acted periodic excitation, when the excitation frequency is two times the natural frequency of beam, divergent oscillation can be found in the transverse direction of the beam. This phenomenon is referred to as dynamic instability. On the other hand, nanobeam structures such as carbon nanotubes (CNTs) are seen as the most promising new material to play an important role in nanotechnology [1,2], and vibration analysis of nano-scale or nano-composite structures has been carried out extensively [3,4]. The dynamic instability of nanobeams remains an open topic in recent years, and the theory of nonlocal continuum elasticity initiated by Eringen [5,6] is usually employed to take into account quantum effects at nanoscale. The major difference between the classical and nonlocal elasticity theories is that the former assumes that stress state at a given point is uniquely affected by strain state at the same point, while the latter considers stress state at a given point as a function of strain states of all points in the continuum.

Reddy [7] applied the nonlocal theory to develop various nonlocal beam models for bending, vibration, and stability of Euler–Bernoulli, Timoshenko, Reddy, and Levinson beams. Subsequently, various mechanical behaviors of nanobeams have been studied by nonlocal beam theories. Arda and Aydogdu [8] investigated the dynamic stability problem of a nanobeam under a time-varying axial loading based on the nonlocal Euler–Bernoulli beam model, which leads to a time-dependent Mathieu-Hill equation. Li et al. [9] studied the dynamics and stability of nonlocal nanobeams by the perturbation method, indicating that nonlocal nanoscale has significant effect on instability region. Ansari et al. [10] analyzed the dynamic stability of embedded single-walled carbon nanotubes (SWCNTs) in a thermal environment based on the nonlocal Bernoulli-Euler and Timoshenko beam theories.

Wang and Li [11] presented the nonlinear primary resonance of nanobeams by nonlocal continuum theory and discussed the influence of Winkler foundation modulus and the ratio of length to diameter. Ansari et al. [12] analyzed the dynamic stability of multi-walled carbon nanotubes (MWCNTs) embedded in a Pasternak-type elastic foundation and a thermal environment based on the nonlocal Timoshenko beam theory, and the dynamic instability regions (DIRs) were obtained by the Bolotin’s method [13]. The method for parametric resonance analysis of an electrically actuated piezoelectric nanobeam resonator was developed by Pourkiaee et al. [14], where surface effects, intermolecular van der Waals forces, and fringing effects are incorporated. Saffari et al. [15] considered the functionally graded form of nanobeams using power law distribution, and obtained the dynamic instability region (DIR) by the nonlocal Timoshenko beam theory.

The dynamic stability analysis of a nonlinear multiple-nanobeam system was conducted by Karličić et al. [16] using the incremental harmonic balance (IHB) method. Huang et al. [17] investigated the dynamic instability of Euler–Bernoulli nanobeams subject to parametric excitation by the Bolotin’s theory, where the matrix singularity problem arising in computing the DIRs was solved and the dynamic responses were computed to verify the accuracy of the boundaries of DIRs. The nonlocal dynamic stability of a Timoshenko nanobeam subjected to a sequence of moving nanoparticles was analyzed by Hashemian et al. [18].

Sourani et al. [19] compared the results of the Bolotin and IHB methods in a dynamic stability analysis of a Euler–Bernoulli nanobeam based on the nonlocal strain gradient theory. Ebrahimi et al. [20] investigated the dynamic instability of Euler–Bernoulli nanobeams under thermo-magneto-mechanical loads based on the nonlocal continuum theory and multiple scales method. Hu et al. [21] studied the dynamic stability of shear deformable nanotubes by the nonlocal strain gradient theory and a size-dependent nonlinear model.

The literature review indicates that, in the existing studies on dynamic stability of nanobeams, the employed nonlocal beam theories mainly include Euler-Bernoulli beams and Timoshenko beams, and the Reddy beam theory has not been applied in the dynamic stability analysis of nanobeams. It is known that for a beam with larger length-to-height ratio, its transverse shear strain can be neglected, and the corresponding beam theory is the Euler-Bernoulli beam theory. When the length-to-height ratio of a beam is so small that its transverse shear strain cannot be ignored, a shear correction factor is used to compensate for the error caused by the constant shear stress assumption. The corresponding beam theory is the Timoshenko beam theory. Considering that the fluctuation of the shear correction factor in variation of length-height-ratio would affect the numerical accuracy, Reddy [22] further proposed a refined theory for beams with a small length-to-height ratio, which accommodates a quadratic variation of transverse shear strain, so it is no longer necessary to use shear correction factor for constant stress assumption. However, the mechanism of dynamical instability analysis of nanobeams by nonlocal Reddy beam theory is still not clear.

Consequently, in this work, the nonlocal Reddy beam theory is applied in the dynamic stability analysis of a simply supported nanobeam embedded in elastic medium, and a Winkler-type elastic foundation is taken to simulate the interaction between the nanobeam and elastic medium. The formulations of dynamic stability based on the nonlocal Reddy beam theory are derived, where the principal dynamic instability regions (PIRs) are obtained by the Bolotin’s method. A parametric study is conducted to investigate the influences of nonlocal elastic parameter, geometric, and material parameters, and the foundation modulus on PIR.

## 2. Mathematical Formulations

### 2.1. Governing Equation of Reddy Beam Embedded in Elastic Matrix

The mechanical behavior of a Reddy beam embedded in an elastic medium has not yet been studied in the literature, so the governing equation of motion is derived in this section.

**Figure 1:**
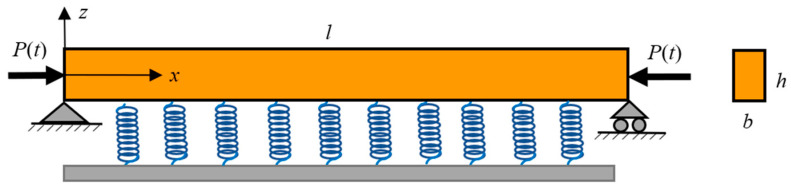
Embedded nonlocal Reddy beam under axial excitation.

A simply supported Reddy-type nanobeam with a rectangular cross-section is illustrated in Figure 1. The nanobeam is embedded in an elastic matrix, and the Winkler foundation modulus is $k_{w}$. The nanobeam is subjected to an axial periodic excitation F(t), where t is the time variable. The length of nanobeam is represented by l, and the width and height of cross-section are denoted by b and h, respectively. For the material properties, Young’s modulus, shear modulus, and mass density are represented by E, G, and ρ, respectively.

A coordinate system (x,y,z) is introduced, where the x-coordinate is taken along the length of the nanobeam, *y*-coordinate along the width of the nanobeam, and z-coordinate along the thickness (the height) of the nanobeam.

The displacement field of the refined Reddy beam can be expressed as [7]

(1) $$u_{1}=u+z\varphi -c_{1}z^{3}\gamma$$

(2) $$u_{2}=0$$

(3) $$u_{3}=w$$

where the displacements $\left(u_{1},u_{2},u_{3}\right)$ denote the displacements along the coordinates (x,y,z); u(x,t) and w(x,t) are the axial and transverse displacements on the mid-plane (z=0) of the beam; φ(x,t) is the rotation of cross-section, and

(4) $$\gamma =\varphi +\frac{\partial w}{\partial x}$$

(5) $$c_{1}=\frac{4}{3h^{2}}$$

The non-zero strains in the Reddy beam can be obtained as

(6) $$\varepsilon_{xx}=\frac{\partial u}{\partial x}+z(1-c_{1}z^{2})\frac{\partial \varphi }{\partial x}-c_{1}z^{3}\frac{\partial^{2}w}{\partial x^{2}}=\varepsilon_{xx}^{0}+zk^{R}+z^{3}\alpha^{R}$$

(7) $$2\varepsilon_{xz}=(1-c_{2}z^{2})\left(\frac{\partial w}{\partial x}+\varphi \right)=\gamma +z^{2}\beta^{R}$$

where $\varepsilon_{xx}$ and $\varepsilon_{xz}$ denote the longitudinal normal strain and the transverse shear strain, respectively.

$\varepsilon_{xx}^{0}$ is the extensional strain

(8) $$\varepsilon_{xx}^{0}=\frac{\partial u}{\partial x}$$

$k^{R}$ is the bending strain of Reddy beam

(9) $$k^{R}=\frac{\partial \varphi }{\partial x}$$

and

(10) $$\alpha^{R}=-c_{1}\left(\frac{\partial \varphi }{\partial x}+\frac{\partial^{2}w}{\partial x^{2}}\right)$$

(11) $$\beta^{R}=-c_{2}\gamma$$

(12) $$c_{2}=\frac{4}{h^{2}}$$

It can be seen from Equation (7) that the transverse shear strain vanishes on the top and bottom faces of the beam (z=±h/2), so it no longer needs a shear correction factor as in the Timoshenko beam theory.

The kinetic energy and strain energy of Reddy beams have the forms of [7,17]

(13) $$T=\frac{1}{2}\int \rho \left[{\left(\frac{\partial u_{1}}{\partial t}\right)}^{2}+{\left(\frac{\partial u_{3}}{\partial t}\right)}^{2}\right]dV$$

(14) $$U=\int_{0}^{l}(N\varepsilon_{xx}^{0}+Mk^{R}+P\alpha^{R}+Q\gamma +R\beta^{R})dx$$

where N, M, P, Q, and R are the stress resultants given in Reddy [7].

The potential energies for the axial excitation and action of the elastic medium are [15,23]

(15) $$V_{F}=-\frac{1}{2}\int_{0}^{l}F{\left(\frac{\partial w}{\partial x}\right)}^{2}dx$$

(16) $$V_{m}=-\frac{1}{2}\int_{0}^{l}F_{m}wdx$$

where $F_{m}=k_{w}w$ is the force from the elastic foundation.

Then, the Hamilton’s principle of axially excited Reddy beams on the Winkler-type elastic foundation can be written as

(17) $$0=\iint \left\{\begin{array}{l} m_{0}\left(\frac{\partial u}{\partial t}\frac{\partial \delta u}{\partial t}+\frac{\partial w}{\partial t}\frac{\partial \delta w}{\partial t}\right)+m_{2}\frac{\partial \varphi }{\partial t}\frac{\partial \delta \varphi }{\partial t}\\ +c_{1}\left[\begin{array}{l} -m_{4}\frac{\partial \varphi }{\partial t}\left(\frac{\partial \delta \varphi }{\partial t}+\frac{\partial^{2}\delta w}{\partial x\partial t}\right)-m_{4}\frac{\partial \delta \varphi }{\partial t}\left(\frac{\partial \varphi }{\partial t}+\frac{\partial^{2}w}{\partial x\partial t}\right)\\ +c_{1}m_{6}\left(\frac{\partial \varphi }{\partial t}+\frac{\partial^{2}w}{\partial x\partial t}\right)\left(\frac{\partial \delta \varphi }{\partial t}+\frac{\partial^{2}\delta w}{\partial x\partial t}\right) \end{array}\right]\\ -N\delta \varepsilon_{xx}^{0}-(M-c_{1}P)\delta k+c_{1}P\frac{\partial^{2}\delta w}{\partial x^{2}}\\ -(Q-c_{2}R)\delta \gamma +k_{w}w\delta w+F\frac{\partial w}{\partial x}\frac{\partial \delta w}{\partial x} \end{array}\right\}dxdt$$

where the constants $m_{i}$ are for a rectangular cross-section nanobeam [24,25]

(18) $$m_{0}=\rho A$$

(19) $$m_{2}=\frac{68}{105}EI+\frac{37}{105}\rho I$$

(20) $$m_{4}=\frac{3}{20}\rho Ih^{2}$$

(21) $$m_{6}=\frac{3}{112}\rho Ih^{4}$$

Equation (17) leads to the Euler-Lagrange equations of motion for the Reddy beam as

(22) $$\begin{array}{l} -m_{0}\frac{\partial^{2}w}{\partial t^{2}}-c_{1}m_{4}\frac{\partial^{3}\varphi }{\partial x\partial t^{2}}+c_{1}^{2}m_{6}\left(\frac{\partial^{3}\varphi }{\partial x\partial t^{2}}+\frac{\partial^{4}w}{\partial x^{2}\partial t^{2}}\right)+\\ c_{1}\frac{\partial^{2}P}{\partial x^{2}}+\frac{\partial (Q-c_{2}R)}{\partial x}-F\frac{\partial^{2}w}{\partial x^{2}}+k_{w}w=0 \end{array}$$

(23) $$-m_{1}\frac{\partial^{2}\varphi }{\partial t^{2}}+c_{1}m_{5}(\frac{\partial^{2}\varphi }{\partial t^{2}}+\frac{\partial^{3}w}{\partial x\partial t^{2}})+\frac{\partial (M-c_{1}p)}{\partial x}-(Q-c_{2}R)=0$$

where

(24) $$m_{1}=\frac{68}{105}EI+\frac{16}{105}\rho I$$

(25) $$m_{5}=\frac{4}{35}\rho Ih^{2}$$

### 2.2. Equation of Motion Based on the Nonlocal Theory

According to the nonlocal theory that the stress at a point in an elastic continuum is the function of strains of all points in the continuum, we have the constitutive relationship in the integral form of nonlocal stress tensor [5,26]

(26) $$\begin{array}{l} \sigma_{ij}(x)=\int \vartheta (\left|x-x^{\prime }\right|,\tau )C_{ijkl}\varepsilon_{kl}(x^{\prime })dV(x^{\prime })\\ \sigma_{ij,j}=0\\ \varepsilon_{ij}=\frac{1}{2}(u_{i,j}+u_{j,i}) \end{array}$$

where $\sigma_{ij}$ and $\varepsilon_{ij}$ are the stress and strain tensors, respectively; $\vartheta (\left|x-x^{\prime }\right|,\tau )$ is the nonlocal modulus function, denoting the nonlocal effect at point x produced by the point at $x^{\prime }$, τ is a material constant affected by internal and external characteristics length, $\tau =e_{0}a/l$, l is the external characteristic length (material length scale parameter), a is the internal characteristic length (crystal lattice parameter), $e_{0}$ is a constant identified from atomic simulation or the dispersion curve of the Born-Karman model using crystal lattice dynamics, $C_{ijkl}$ is the elasticity modulus tensor of classical isotropic elasticity, and $u_{i}$ is the displacement vector.

The integral form of the nonlocal constitutive relationship is complicated and it has the simpler differential form for unidimensional nanobeams

(27) $$\sigma_{xx}-\mu \frac{\partial^{2}\sigma_{xx}}{\partial x^{2}}=E\varepsilon_{xx}$$

(28) $$\sigma_{xz}-\mu \frac{\partial^{2}\sigma_{xz}}{\partial x^{2}}=2G\varepsilon_{xz}$$

where $\mu ={\left(e_{0}a\right)}^{2}$ is the nonlocal parameter.

Substituting Equations (27) and (28) and Equations (6) and (7) into the expressions of N, M, P, Q, and R, we obtain the following force-strain relations for the nonlocal Reddy beam

(29) $$M-\mu \frac{\partial^{2}M}{\partial x^{2}}=EIk^{R}+EJ\alpha^{R}$$

(30) $$P-\mu \frac{\partial^{2}P}{\partial x^{2}}=EJk^{R}+EK\alpha^{R}$$

(31) $$Q-\mu \frac{\partial^{2}Q}{\partial x^{2}}=GA\gamma +GI\beta^{R}$$

(32) $$R-\mu \frac{\partial^{2}R}{\partial x^{2}}=GI\gamma +GJ\beta^{R}$$

where $(A,I,J,K)=\int_{A}(1,z^{2},z^{4},z^{6})dA$ are the second, fourth, and sixth order moments of the area around the y-axis.

Further substituting Equations (29) to (32) into Equations (22) and (23) leads to the equation of motion for the nonlocal Reddy nanobeam:

(33) $$\begin{array}{l} G\tilde{A}(\frac{\partial \varphi }{\partial x}+\frac{\partial^{2}w}{\partial x^{2}})-F\frac{\partial^{2}w}{\partial x^{2}}+k_{w}w+\mu \frac{\partial^{2}}{\partial x^{2}}(F\frac{\partial^{2}w}{\partial x^{2}}-k_{w}w)\\ +c_{1}EJ\frac{\partial^{3}\varphi }{\partial x^{3}}-c_{1}{}^{2}EK\left(\frac{\partial^{3}\varphi }{\partial x^{3}}+\frac{\partial^{4}w}{\partial x^{4}}\right)\\ =m_{0}\frac{\partial^{2}w}{\partial t^{2}}+c_{1}m_{4}\frac{\partial^{3}\varphi }{\partial x\partial t^{2}}-c_{1}{}^{2}m_{6}\left(\frac{\partial^{3}\varphi }{\partial x\partial t^{2}}+\frac{\partial^{4}w}{\partial x^{2}\partial t^{2}}\right)\\ -\mu \left[m_{0}\frac{\partial^{4}w}{\partial x^{2}\partial t^{2}}+c_{1}m_{4}\frac{\partial^{5}\varphi }{\partial x^{3}\partial t^{2}}-c_{1}{}^{2}m_{6}\left(\frac{\partial^{5}\varphi }{\partial x^{3}\partial t^{2}}+\frac{\partial^{6}w}{\partial x^{4}\partial t^{2}}\right)\right] \end{array}$$

(34) $$\begin{array}{l} E\hat{I}\frac{\partial^{2}\varphi }{\partial x^{2}}-c_{1}E\hat{J}(\frac{\partial^{2}\varphi }{\partial x^{2}}+\frac{\partial^{3}w}{\partial x^{3}})-G\tilde{A}(\varphi +\frac{\partial w}{\partial x})\\ =m_{1}\frac{\partial^{2}\varphi }{\partial t^{2}}-c_{1}m_{5}(\frac{\partial^{2}\varphi }{\partial t^{2}}+\frac{\partial^{3}w}{\partial x\partial t^{2}})\\ -\mu \left[m_{1}\frac{\partial^{4}\varphi }{\partial x^{2}\partial t^{2}}-c_{1}m_{5}\left(\frac{\partial^{4}\varphi }{\partial x^{2}\partial t^{2}}+\frac{\partial^{5}w}{\partial x^{3}\partial t^{2}}\right)\right] \end{array}$$

where the constants are [7]

(35) $$\tilde{A}=\frac{8}{15}A$$

(36) $$\hat{I}=\frac{4}{5}I$$

(37) $$\hat{J}=\frac{4}{35}Ih^{2}$$

### 2.3. Governing Equation of Dynamic Instability

For a simply supported nanobeam, its displacements w(x,t) and φ(x,t) can be expressed as

(38) $$w(x,t)=\Lambda (t)\sin(\frac{n\pi x}{l})$$

(39) $$\varphi (x,t)=\chi (t)\cos(\frac{n\pi x}{l})$$

where *n* denotes the number of modes.

Substituting Equations (38) and (39) into Equations (33) and (34) gives

(40) $$\left\{\begin{array}{l} -G\tilde{A}\left[\frac{n\pi }{l}\chi +{\left(\frac{n\pi }{l}\right)}^{2}\Lambda \right]+F{\left(\frac{n\pi }{l}\right)}^{2}\Lambda +k_{w}\Lambda +\mu \left[F{\left(\frac{n\pi }{l}\right)}^{4}+k_{w}{\left(\frac{n\pi }{l}\right)}^{2}\right]\Lambda \\ +c_{1}EJ{\left(\frac{n\pi }{l}\right)}^{3}\chi -c_{1}{}^{2}EK{\left(\frac{n\pi }{l}\right)}^{3}\chi -c_{1}{}^{2}EK{\left(\frac{n\pi }{l}\right)}^{4}\Lambda \\ -m_{0}\ddot{\Lambda }+c_{1}m_{4}\frac{n\pi }{l}\ddot{\chi }-c_{1}{}^{2}m_{6}\left[\frac{n\pi }{l}\frac{\partial^{2}\chi }{\partial t^{2}}+{\left(\frac{n\pi }{l}\right)}^{2}\ddot{\Lambda }\right]\\ -\mu \left[m_{0}{\left(\frac{n\pi }{l}\right)}^{2}\ddot{\Lambda }-c_{1}m_{4}{\left(\frac{n\pi }{l}\right)}^{3}\ddot{\chi }+c_{1}{}^{2}m_{6}{\left(\frac{n\pi }{l}\right)}^{3}\ddot{\chi }+c_{1}{}^{2}m_{6}{\left(\frac{n\pi }{l}\right)}^{4}\ddot{\Lambda }\right] \end{array}\right\}\sin\left(\frac{n\pi x}{l}\right)=0$$

(41) $$\left\{\begin{array}{l} -E\hat{I}{\left(\frac{n\pi }{l}\right)}^{2}\chi +c_{1}E\hat{J}\left[{\left(\frac{n\pi }{l}\right)}^{2}\chi +{\left(\frac{n\pi }{l}\right)}^{3}\Lambda \right]-G\tilde{A}\left[\chi +\frac{n\pi }{l}\Lambda \right]\\ -m_{1}\ddot{\chi }+c_{1}m_{5}\left[\ddot{\chi }+\frac{n\pi }{l}\ddot{\Lambda }\right]-\mu m_{1}{\left(\frac{n\pi }{l}\right)}^{2}\ddot{\chi }+\mu c_{1}m_{5}{\left(\frac{n\pi }{l}\right)}^{2}\ddot{\chi }+\mu c_{1}m_{5}{\left(\frac{n\pi }{l}\right)}^{3}\cos\ddot{\Lambda } \end{array}\right\}\cos\left(\frac{n\pi x}{l}\right)=0$$

where the dot superscript indicates the derivation with respect to time.

In order to ensure that Equations (38) and (39) really satisfy Equations (33) and (34), it is necessary and sufficient that the quantity in the brackets before $\sin\left(n\pi x/l\right)$ and $\cos\left(n\pi x/l\right)$ should vanish at any time t, which can be written in matrix form

(42) $$M\ddot{d}+\left[K_{e}-F(t)K_{g}\right]d=0$$

where

(43) $$d=\left\{\begin{array}{c} \Lambda (t)\\ \chi (t) \end{array}\right\}$$

is displacement vector,

(44) $$M=\left[\begin{array}{cc} a_{1} & a_{2}\\ a_{3} & a_{4} \end{array}\right]$$

is mass matrix,

(45) $$K_{e}=\left[\begin{array}{cc} a_{5} & a_{6}\\ a_{7} & a_{8} \end{array}\right]$$

is stiffness matrix and

(46) $$K_{g}=\left[\begin{array}{cc} a_{9} & 0\\ 0 & 0 \end{array}\right]$$

is geometric stiffness matrix, with

(47) $$a_{1}=-m_{0}-(c_{1}{}^{2}m_{6}+\mu m_{0}){\left(\frac{n\pi }{l}\right)}^{2}-\mu c_{1}{}^{2}m_{6}{\left(\frac{n\pi }{l}\right)}^{4}$$

(48) $$a_{2}=(c_{1}m_{4}-c_{1}{}^{2}m_{6})\frac{n\pi }{l}+\mu (c_{1}m_{4}-c_{1}{}^{2}m_{6}){\left(\frac{n\pi }{l}\right)}^{3}$$

(49) $$a_{3}=c_{1}m_{5}\left[\frac{n\pi }{l}+\mu {\left(\frac{n\pi }{l}\right)}^{3}\right]$$

(50) $$\begin{array}{l} a_{4}=-m_{1}+c_{1}m_{5}-\mu {\left(\frac{n\pi }{l}\right)}^{2}(m_{1}-c_{1}m_{5}) \end{array}$$

(51) $$a_{5}=k_{w}-{\left(\frac{n\pi }{l}\right)}^{2}(G\tilde{A}-\mu k_{w})-{\left(\frac{n\pi }{l}\right)}^{4}c_{1}{}^{2}EK$$

(52) $$a_{6}=-G\tilde{A}\frac{n\pi }{l}+(c_{1}EJ-c_{1}{}^{2}EK){\left(\frac{n\pi }{l}\right)}^{3}$$

(53) $$a_{7}=-G\tilde{A}\frac{n\pi }{l}+c_{1}E\hat{J}{\left(\frac{n\pi }{l}\right)}^{3}$$

(54) $$a_{8}=-G\tilde{A}+(c_{1}E\hat{J}-E\hat{I}){\left(\frac{n\pi }{l}\right)}^{2}$$

(55) $$a_{9}=-{\left(\frac{n\pi }{l}\right)}^{2}-\mu {\left(\frac{n\pi }{l}\right)}^{4}$$

When the axial excitation is static, the term of inertia force in Equation (42) disappears, and Equation (42) is reduced to the problem of static instability:

(56) $$\left|K_{e}-F_{cr}K_{g}\right|=0$$

where $F_{cr}$ is Euler critical load.

By disregarding axial excitation and considering dynamic displacement vector as $d=\tilde{d}e^{i\omega t}$, Equation (42) degenerates into the free vibration problem

(57) $$\left|K_{e}-\omega^{2}M\right|=0$$

where ω denotes the natural frequency of the Reddy nanobeam.

When F(t) is periodically time dependent, it can be defined as

(58) $$F(t)=(\alpha +\beta \cos\theta t)F_{cr}$$

where α and β denote the ratios of mean and amplitude of F(t) with respect to Euler load, and θ is the excitation frequency.

Substituting Equation (58) into Equation (42) results in

(59) $$M\ddot{d}+\left[K_{e}-(\alpha +\beta \cos\theta t)F_{cr}K_{g}\right]d=0$$

Equation (59) is a second-order differential equation with periodic coefficients, called the Mathieu-Hill equation. The solutions of the Mathieu-Hill equation, i.e., the dynamic responses of the Reddy nanobeam, can be categorized into those that are stable and those that are unstable, and these solutions cluster together to form the stable and unstable regions, respectively. The regions of stable solutions correspond to dynamic stability state of the nanobeam, so they are called the dynamic stability regions (DSRs). Meanwhile, the regions of unstable solutions correspond to dynamic instability of the nanobeam and they are named the dynamic instability regions (DIRs). Moreover, the boundaries between DSRs and DIRs are determined by the periodic solutions of the Mathieu-Hill equation with the periods of T and 2T (T=2π/θ). Each period T or 2T corresponds to a series of DIRs, and the first DIRs under period 2T has the largest width and is the most dangerous region, which is called the principal dynamic instability region (PIR).

The boundary of the PIR can be determined by solving the following eigenvalue problems [13,15]:

(60) $$\left|K_{e}-(\alpha \pm \frac{\beta }{2})F_{cr}{Κ}_{g}-\frac{\theta^{2}}{4}M\right|=0$$

Since Equation (60) is identical for all n modes, the index n can be dropped in solving Equation (60). For a deterministic nanobeams, matrices $K_{e}$, ${Κ}_{g}$, and M and Euler load $F_{cr}$ are fixed, and the corresponding excitation frequency θ can be obtained under the given α and β. Thus, boundaries of the PIR can be depicted on a three-dimensional parametric space enclosed by α, β, and θ. Excitation parameter e=β/[2(1−α)] used in the classic Bolotin’s method and nondimensionalized excitation frequency $\bar{\theta }=\theta L\sqrt{\rho /E}$ can be further employed for conveniently demonstrating the PIR on a two-dimensional parametric plane $\left(\bar{\theta },e\right)$ [17,19].

## 3. Results and Discussions

**Figure 2:**
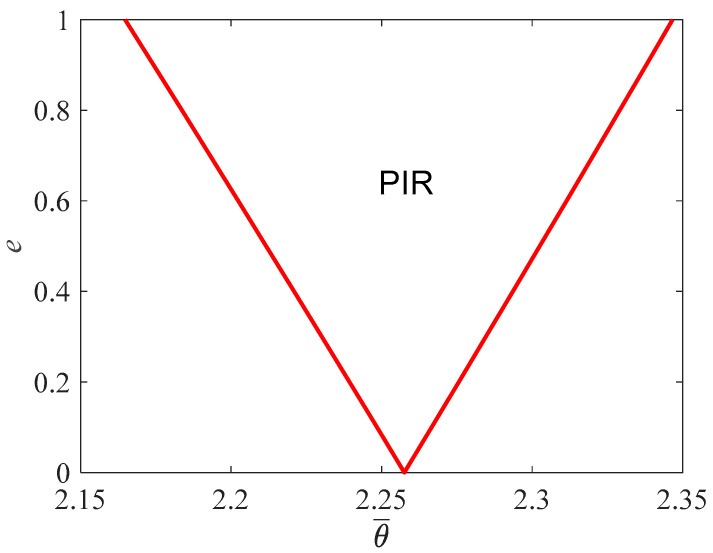
Boundary of PIR of the Reddy nanobeam.

Material and geometric parameters of the studied nonlocal Reddy beam are listed in Table 1. The corresponding Euler load and natural frequency are 1.15 × 10^−8^ N, and 6.83 × 10^12^ Hz, respectively, and the boundary of the PIR is given in Figure 2. According to Figure 2, the dimensionless excitation frequency is $\bar{\theta }=2.26$ under *e* = 0, i.e., the excitation frequency is $\theta =13.16\times 10^{12}$ Hz, which is about two times the natural frequency.

As shown in Figure 2, when the excitation parameter is within 1.0, the Reddy nanobeam might become dynamically unstable when the dimensionless excitation frequency is in the range of [2.165, 2.347]. If the point $\left(\bar{\theta },e\right)$ is located inside the PIR, the corresponding dynamic response takes on the currency of dynamic instability. Otherwise, when the point $\left(\bar{\theta },e\right)$ is outside the PIR, the dynamic response is stable with time.

The mathematical formulations in Section 2 have indicated that the boundary of the PIR is controlled by the geometric and material parameters shown in Table 1. In the following, the effects of each parameter on the boundary of the PIR are investigated. For the investigated parameter, other parameters in Table 1 are kept invariant.

**Figure 3:**
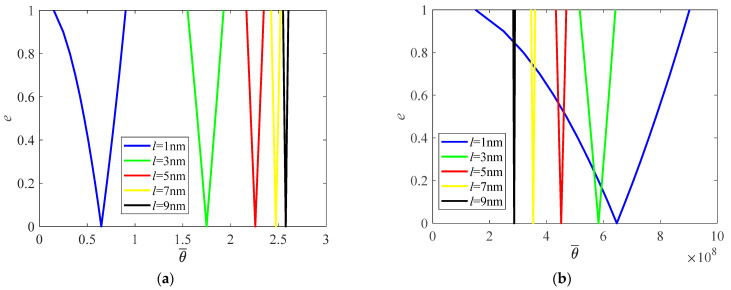
PIRs under various lengths of Reddy nanobeam. (**a**) $\bar{\theta }=\theta L\sqrt{\rho /E}$; (**b**) $\bar{\theta }=\theta \sqrt{\rho /E}$.

Comparison of PIRs under various lengths of the Reddy nanobeam is shown in Figure 3. It is indicated that the PIR shifts to the zone with higher frequency of excitation when the length increases and excitation frequency is nondimensionalized by $\bar{\theta }=\theta L\sqrt{\rho /E}$ (Figure 3a). In order to eliminate the influence of length during nondimensionalization, excitation frequency is rescaled by $\bar{\theta }=\theta \sqrt{\rho /E}$ and the PIRs are redrawn in Figure 3b. It is demonstrated that the PIR moves to the zone with lower excitation frequency when the length increases, and the width of the PIR decreases significantly.

**Figure 4:**
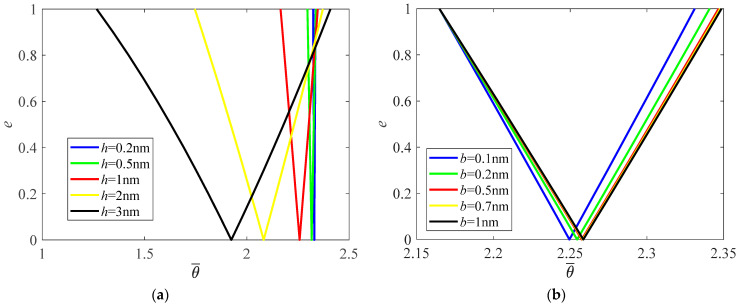
Effects of sectional dimensions on PIRs. (**a**) Height; (**b**) Width.

When the height of the cross-section of the nanobeam increases and $\bar{\theta }=\theta L\sqrt{\rho /E}$, PIR also shifts to a lower frequency zone, and the width of the PIR increases obviously (Figure 4a). However, sectional width has a small impact on the position and width of the PIR (Figure 4b).

**Figure 5:**
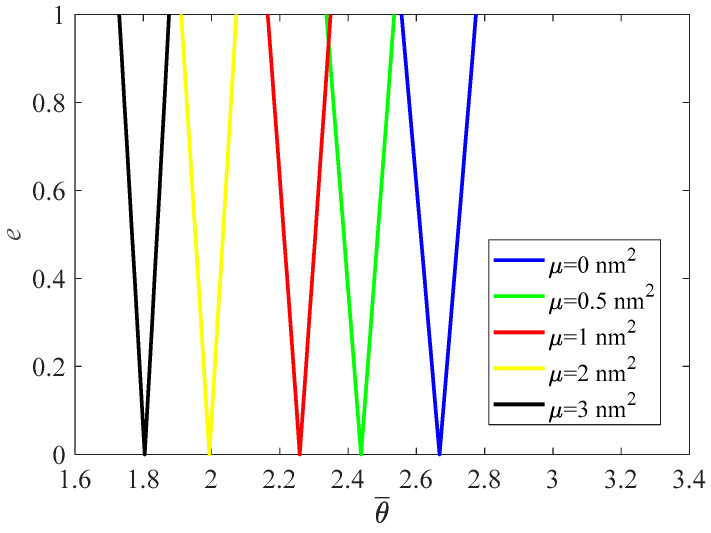
PIRs under various nonlocal parameter.

Figure 5 demonstrates that, with the increase in the nonlocal parameter, PIR shifts to a lower frequency zone while the width of the PIR is slightly reduced ($\bar{\theta }=\theta L\sqrt{\rho /E}$). Therefore, the local beam model (μ=0) would overestimate the frequency and width of the PIR.

**Figure 6:**
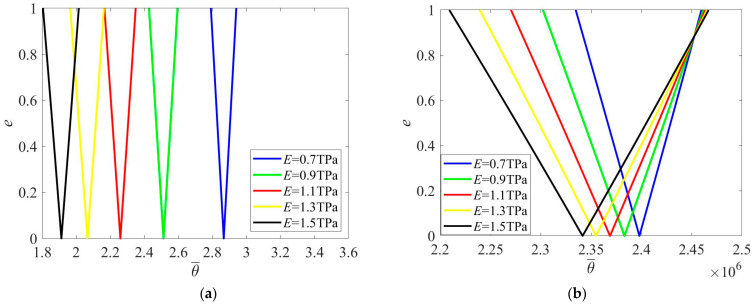
PIRs under various Young’s modulus. (**a**) $\bar{\theta }=\theta L\sqrt{\rho /E}$; (**b**) $\bar{\theta }=\theta L\sqrt{\rho }$.

With the increase in Young’s modulus, the PIR also shifts to a lower frequency zone when excitation frequency is nondimensionalized by $\bar{\theta }=\theta L\sqrt{\rho /E}$ (Figure 6a). For eliminating the effect of Young’s modulus in the nondimensionalization, excitation frequency is rescaled by $\bar{\theta }=\theta L\sqrt{\rho }$ in Figure 6b. It is shown that width of the PIR increases when Young’s modulus is raised.

**Figure 7:**
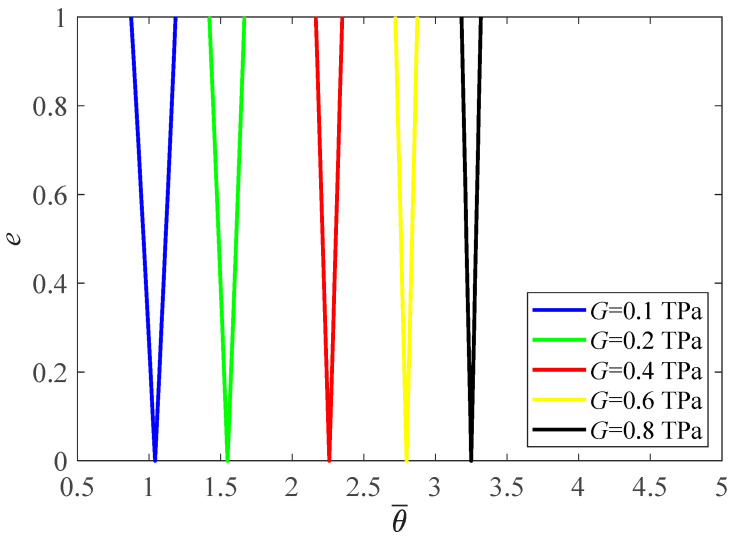
PIRs under various shear modulus.

In contrary to Young’s modulus, the increment of shear modulus makes the PIR move to a higher frequency zone (Figure 7, $\bar{\theta }=\theta L\sqrt{\rho /E}$).

**Figure 8:**
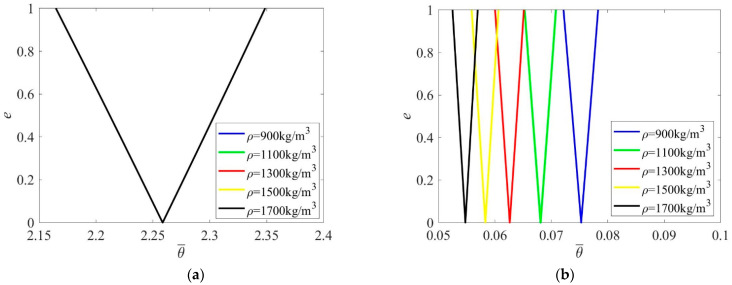
PIRs under different mass densities. (**a**) $\bar{\theta }=\theta L\sqrt{\rho /E}$; (**b**) $\bar{\theta }=\theta L/\sqrt{E}$.

Mass density has very few effects on the PIR when excitation frequency is nondimensionalized by $\bar{\theta }=\theta L\sqrt{\rho /E}$ (Figure 8a), but mass density makes the PIR move to a lower frequency zone when excitation frequency is zoomed by $\bar{\theta }=\theta L/\sqrt{E}$ (Figure 8b).

**Figure 9:**
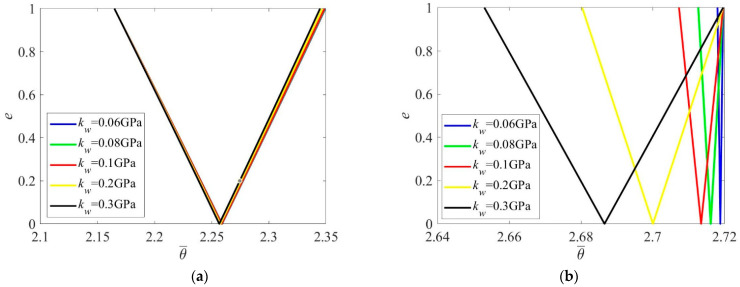
PIRs under various foundation modulus ($\bar{\theta }=\theta L\sqrt{\rho /E}$). (**a**) l=5nm; (**b**) l=20nm.

As illustrated in Figure 9a, foundation modulus has limited influence on the PIR when the nanobeam is short. However, with increase in length, the effect of foundation modulus becomes more remarkable, where the PIR moves to a lower frequency zone and its width becomes larger (Figure 9b). Hence, elasticity of foundation would aggravate the dynamic stability of Reddy nanobeams.

## 4. Conclusions

Considering that the dynamic instability of nonlocal Reddy beams has not yet been investigated, this study carefully derives the governing equations of motion and dynamic stability for a simply supported Reddy nanobeam embedded in elastic medium, based on the nonlocal theory and Reddy’s beam theory.

The study demonstrates that the increase in length of a Reddy nanobeam makes the principal instability region (PIR) move to a lower frequency zone and the width of the PIR shrink obviously. Increase in sectional height of the nanobeam also causes a shift of the PIR to a lower frequency zone, but the width of the PIR enlarges significantly. Sectional width has few effects on the PIR. Under a larger nonlocal parameter, the PIR shifts to a lower frequency zone while the width of PIR is less influenced.

When the Young’s modulus of the nanobeam increases, the PIR moves to a lower frequency zone and the width of the PIR enlarges. On the contrary, with increase in shear modulus, the PIR moves to a higher frequency zone and the width of the PIR shrinks. When mass density increases, the PIR moves to a lower frequency zone. Foundation modulus also has few effects on the PIR under shorter nanobeams, but the effect becomes significant when the nanobeam becomes longer.

## Figures and Tables

**Table 1:** Parameters of the studied nanobeam.

Material Parameters		Geometric Parameter	
*E* (TPa)	1.1	*l* (nm)	5
*ρ* (kg/m^3^)	1300	*h* (nm)	1
*G* (TPa)	0.4	*b* (nm)	0.5
*k_w_* (GPa)	0.1	*μ* (nm^2^)	1

## Data Availability

The research data can be found in the paper.
